# Dopamine Modulates Dynamic Decision-Making during Foraging

**DOI:** 10.1523/JNEUROSCI.2586-19.2020

**Published:** 2020-07-01

**Authors:** Campbell Le Heron, Nils Kolling, Olivia Plant, Annika Kienast, Rebecca Janska, Yuen-Siang Ang, Sean Fallon, Masud Husain, Matthew A.J. Apps

**Affiliations:** ^1^Nuffield Department of Clinical Neurosciences, University of Oxford, Oxford OX39DU, United Kingdom; ^2^New Zealand Brain Research Institute, Christchurch 8011, New Zealand; ^3^Department of Medicine, University of Otago, Christchurch 8011, New Zealand; ^4^Department of Experimental Psychology, University of Oxford, Oxford OX2 6GG, United Kingdom; ^5^Wellcome Centre for Integrative Neuroimaging, University of Oxford, Oxford OX3 9DU, United Kingdom; ^6^Bristol Medical School, University of Bristol, Bristol BS8 1UD, United Kingdom; ^7^Centre for Human Brain Health, School of Psychology, University of Birmingham, Birmingham, B15 2TT, United Kingdom

**Keywords:** decision making, dopamine, foraging, opportunity cost, reward

## Abstract

The mesolimbic dopaminergic system exerts a crucial influence on incentive processing. However, the contribution of dopamine in dynamic, ecological situations where reward rates vary, and decisions evolve over time, remains unclear. In such circumstances, current (foreground) reward accrual needs to be compared continuously with potential rewards that could be obtained by traveling elsewhere (background reward rate), to determine the opportunity cost of staying versus leaving. We hypothesized that dopamine specifically modulates the influence of background, but not foreground, reward information when making a dynamic comparison of these variables for optimal behavior. On a novel foraging task based on an ecological account of animal behavior (marginal value theorem), human participants of either sex decided when to leave locations in situations where foreground rewards depleted at different rates, either in rich or poor environments with high or low background reward rates. In line with theoretical accounts, people's decisions to move from current locations were independently modulated by changes in both foreground and background reward rates. Pharmacological manipulation of dopamine D2 receptor activity using the agonist cabergoline significantly affected decisions to move on, specifically modulating the effect of background reward rates. In particular, when on cabergoline, people left patches in poor environments much earlier. These results demonstrate a role of dopamine in signaling the opportunity cost of rewards, not value per se. Using this ecologically derived framework, we uncover a specific mechanism by which D2 dopamine receptor activity modulates decision-making when foreground and background reward rates are dynamically compared.

**SIGNIFICANCE STATEMENT** Many decisions, across economic, political, and social spheres, involve choices to “leave”. Such decisions depend on a continuous comparison of a current location's value, with that of other locations you could move on to. However, how the brain makes such decisions is poorly understood. Here, we developed a computerized task, based around theories of how animals make decisions to move on when foraging for food. Healthy human participants had to decide when to leave collecting financial rewards in a location, and travel to collect rewards elsewhere. Using a pharmacological manipulation, we show that the activity of dopamine in the brain modulates decisions to move on, with people valuing other locations differently depending on their dopaminergic state.

## Introduction

The mesolimbic dopaminergic system plays a crucial role in motivating behavior and has been closely linked to neural circuits which convey information about incentives (Schultz and Dickinson, 2000; Haber and Knutson, 2010; Salamone and Correa, 2012; Hamid et al., 2016). Several experiments across species have demonstrated a crucial role for dopamine in overcoming costs to obtain rewards (Salamone and Correa, 2012; Le Bouc et al., 2016; Syed et al., 2016; Le Heron et al., 2018b) and for learning about rewarding outcomes to update future behavior (Pessiglione et al., 2006; Schultz, 2016). Tasks probing dopamine function typically use a bandit-style design, with choices made between presented options, each associated with a value that is learned by trial and error (Salamone et al., 2007; Schultz, 2016; Le Heron et al., 2018b). Yet, in real-world settings, many of our decisions are not simply choices between discreet stimuli. Moreover, animal models increasingly highlight that dopamine signals change gradually as the rate of obtaining rewards changes, suggesting a need to examine dopamine's role in settings where rewards are dynamically accrued (Howe et al., 2013; Hamid et al., 2016; Mohebi et al., 2019).

One real-world dynamic decision is whether to stay in a location or switch to an alternative to maximize rewards (Pearson et al., 2014; Mobbs et al., 2018). Such decision-making requires a continuous comparison between the current rate of reward accumulation at a location with the average reward rate available in the environment to guide leaving behavior- often referred to as foreground and background, respectively, in behavioral ecology (Rutledge et al., 2009; Kurniawan et al., 2011; Constantino and Daw, 2015). However, despite the clear ecological significance of reward rate comparisons for decisions to move on, only one study has examined the role of dopamine in modulating human decisions to leave (Constantino et al., 2017).

It has been proposed that tonic (slower-changing) dopamine signals encode information about background reward rates (Niv et al., 2007). Voltammetry experiments in rodents have linked slow changes in dopamine levels to the average rate of reward intake (Hamid et al., 2016). Motor vigor changes in humans as a function of average reward rate and dopaminergic manipulations (Beierholm et al., 2013; Guitart-Masip et al., 2014; Le Bouc et al., 2016; Le Heron et al., 2018b). In a recent study, Parkinson's disease patients' decisions to stay or leave when collecting rewards were influenced by average reward rates, which depended on medication state (Constantino et al., 2017). However, it is unclear whether this extends to leaving decisions in healthy people when reward rates are continuous and dynamic.

Marginal Value Theorem (MVT), a model from behavioral ecology that predicts many species' foraging behaviors (Nonacs, 2001; Stephens and Brown, 2007), provides a formal framework for how to decide to leave a location (“patch”) as rewards dynamically deplete (Charnov, 1976; Stephens and Krebs, 1986). It states that animals should continuously compare the instantaneous foreground reward rate with the average background reward rate, and an optimal forager leaves when the former falls below the latter (Charnov, 1976; Stephens and Krebs, 1986; Pearson et al., 2014). According to MVT, these two rates independently impact when to leave, making this framework ideal for testing whether dopamine processes background reward rates. Although recent work has suggested humans can learn how to maximize rewards in a “patch-leaving” context (Constantino and Daw, 2015), still relatively little is known about whether MVT principles extend to human behavior and whether they depend on dopaminergic activity (Hutchinson et al., 2008; Pearson et al., 2014; Mobbs et al., 2018; Gabay and Apps, 2020).

We developed a novel ecologically derived task in which participants chose when to move on as foreground and background reward rates varied. We hypothesized that both young and old human participants would make leaving decisions in accordance with MVT, and that manipulating dopamine receptor activity using the D2-agonist cabergoline would selectively modulate the influence of background reward rate on decisions to move on.

## Materials and Methods

To test the hypothesis that manipulating dopamine availability would modulate the influence of background reward rates on patch-leaving decisions in humans, we designed a novel foraging-based task. In the first study, we highlight the validity of this task in healthy young participants. In the second, we manipulated dopamine availability pharmacologically, testing the influence of cabergoline administration on older adults in a double-blind, placebo-matched, crossover design.

### 

#### Participants

This study was approved by the local research ethics committee, and written informed consent was obtained from all participants.

##### Study 1

Forty healthy volunteers (mean age 24, range 20–30 years) of both genders were recruited via a local database. One was subsequently excluded because of poor engagement with the task (identified at debriefing).

##### Study 2

Thirty healthy older (mean age 69, range 60–78 years) participants of both genders were recruited via a local database. Potential participants were screened for the presence of neurologic, psychiatric, or cardiovascular diseases, or for the use of medications that could interact with cabergoline, and excluded if any of these were present. One subject was subsequently excluded because a core metric of task performance (variance in leaving times per condition) fell outside 3 SDs of the mean variance, leaving 29 participants for analysis.

#### Experimental design

**Figure 1.**
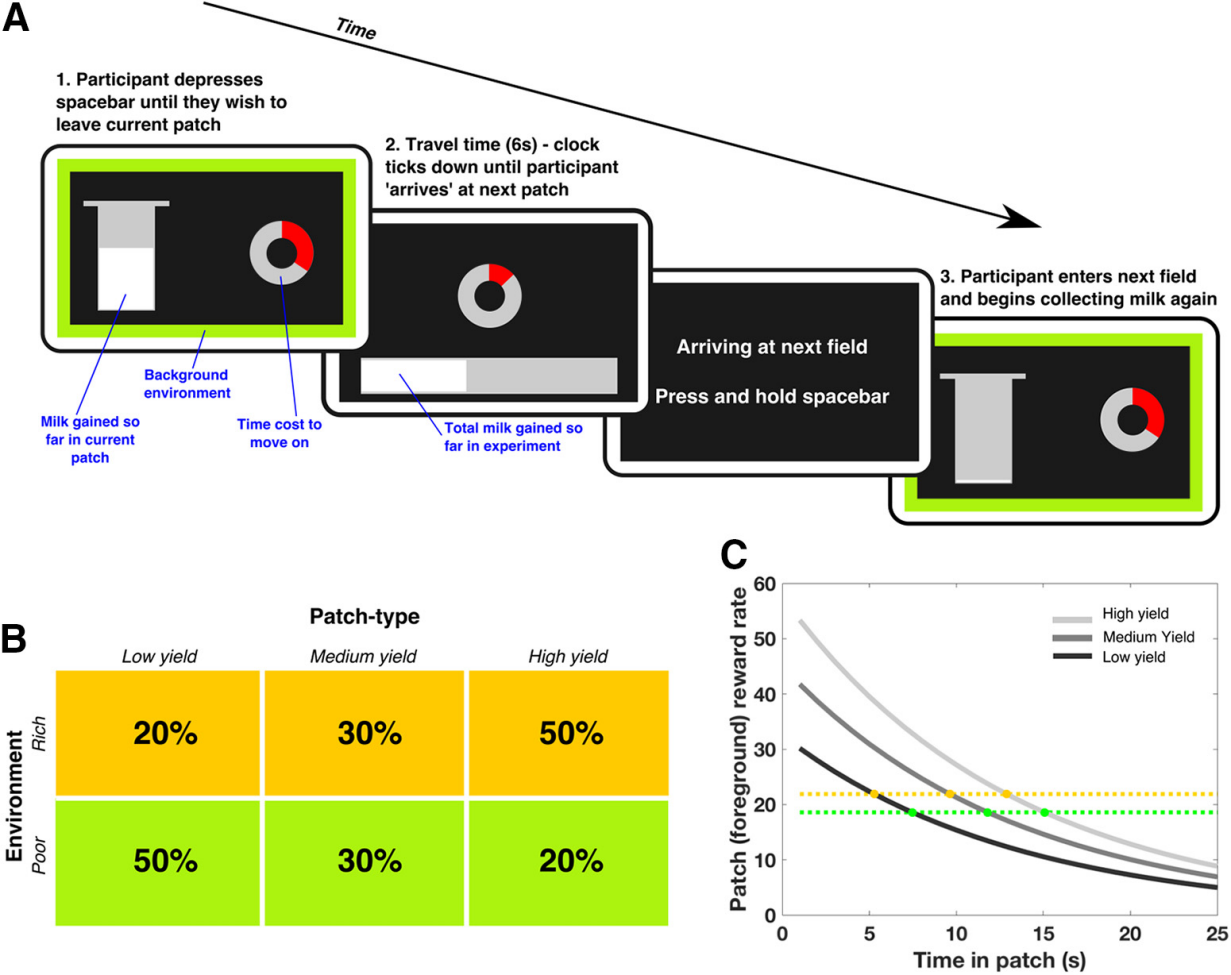
Patch leaving paradigm. ***A***, Participants had to decide how long to remain in their current patch (field), in which reward (milk) was returned at an exponentially decreasing rate (displayed on the screen by continuous filling [white bar] of the silver bucket), before moving on to the next patch, which incurred a fixed cost of 6 s during which they could collect no reward. Their goal was to maximize milk return across the whole experiment. The instantaneous rate of bucket filling indicated the foreground reward rate, whereas the colored frame indicated the distribution of different patch types and their average value, and thus the background reward rate. Participants were aware they had ∼10 min in each environment (which were blocked), but were not shown any cues to indicate how much total time had elapsed. Following a leave decision, a clock ticking down the 6 s travel time was presented. ***B***, Three foreground patch types were used, differing in the scale of filling of the milk bucket (low, medium, and high yield), which determined the foreground reward rate. Two different background environments (farms) were used, with the background reward rate determined by the relative proportions of these patch types. The rich environment contained a higher proportion of high-yield fields, and a lower proportion of low-yield ones, meaning it had a higher background reward rate than the green farm, which had a higher proportion of low-yield fields. ***C***, According to MVT, participants should leave each patch when the instantaneous reward rate in that patch (gray lines) drops to the background environmental average (gold and green dotted lines). Therefore, people should leave sooner from all patches in rich (gold dotted line) compared with poor (green dotted line) environments, but later in high-yield compared with low-yield patches. Crucially, these two effects are independent from each other.

All participants were administered a computer-based patch-leaving task in which they had to decide when to move on from a current patch. The task design independently manipulated background and foreground reward rates, based on the principles of MVT, a theory of optimal foraging behavior (Charnov, 1976; Stephens and Krebs, 1986). The task was framed as a farming game in which people had to collect as much milk (reward) as possible; this would be sold at a market at the end of the game, and their financial remuneration was according to the milk accrued. Participants spent a fixed time in each of two farms, presented in a blocked design, with order pseudorandomized across subjects, collecting milk from fields of cows and making decisions of whether to move on (leave the current field for the next one) (Fig. 1*A*). Moving on to the next field incurred a time cost (travel time) during which no milk could be collected.

Participants aimed to maximize their overall reward returns by deciding how long to spend in these sequentially encountered patches, in which the current (foreground) reward rate decreased in an exponential manner. The reward obtained so far in the patch was displayed as a bucket which continuously filled during patch residency.

The foreground reward rate can be manipulated by several factors, one of which is the patch “quality,” (yield, or density) of rewards available. The higher the yield, the higher the initial rate of reward will be obtained which, if all other properties are equal, leads to the foreground reward rate taking longer to reach the background reward rate. Thus, higher patch quality equates to a prediction of a longer residency time. Three patch types were used, differing in the scaling factor of the reward function (***S*** in Eq. 1 below), and corresponding to low (32.5), medium (45), and high (57.5) yield patches. The foreground reward rate, after ***T*** seconds in a patch, was determined by the following equation:

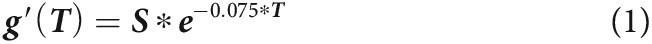


The height of milk displayed in the bucket was proportional to the integral of Equation 1 between time = 0 and T, and was updated with a frequency of 20 Hz. We aimed to change the foreground reward rate by manipulating patch quality. Importantly, participants were not explicitly instructed which patch type they were currently in; rather, they could only infer this by observing the rate at which the milk pail filled. As such, participants had to monitor the foreground reward to know the quality of the patch.

The background reward rate was manipulated by varying the proportions of low, medium, and high yield patches in “farms,” in a pseudorandomized fashion (Fig. 1*B*). In the rich farm (environment), 50% of the patches were high yield, 30% medium, and 20% low yield, while in the poor farm 50% of the patches were low yield, 30% medium, and 20% high. Therefore, the background reward rate was higher in the rich environment. The background reward rate during each block was continuously cued by the colored border on the screen, indicating either the rich (gold border) or poor farm (green border). MVT demonstrates that, to maximize reward gain, participants should leave each field when the instantaneous reward rate in the field (from Eq. 1) drops below the background average reward rate for the farm. Simply, for a given patch type, participants should leave earlier in the rich environment compared with the poor environment (Fig. 1*C*).

#### Procedure

Before commencing the experiment, participants were trained on the task elements using a structured explanation and practice session lasting ∼20 min. During the practice session, participants encountered 10 patches in the rich farm, and then 10 in the poor farm, making a patch-leaving decision from each, and each time having to wait the 6 s transit time before beginning to accumulate milk in the next patch. Patches in each farm type were presented in a pseudo-random order such that the final encountered ratios were as displayed in Figure 1*B*. Comprehension of the different elements was checked verbally before commencing the main experiment, with volunteers asked to explain what each display item meant. They were not given any instructions as to what optimal behavior would be. However, they were told they would spend an equal amount of time on the two farm types (gold and green) and that they would never run out of fields. Participants were seated in front of a desktop computer running Pyschtoolbox (http://psychtoolbox.org/) implemented within MATLAB (The MathWorks).

When participants chose to leave their current patch (by releasing the spacebar they had been holding down), they incurred a fixed time cost of 6 s, described as the time to walk to the next patch. During this time, a counter was displayed which ticked down the seconds until the next patch was reached. On arriving at the next patch, participants were cued to “press and hold the spacebar,” and after doing this the screen display changed to show the new patch.

##### Study 1

Participants were tested in a single session following training as above.

##### Study 2

This was conducted as a randomized, double-blind, placebo-controlled study. Participants were tested in two separate sessions: once following administration of a single dose of 1 mg cabergoline (which stimulates postsynaptic D2 receptors) (Brooks et al., 1998) and once following administration of an indistinguishable placebo tablet. An older population was chosen because they may have a relative dopaminergic deficit compared with younger people (Karrer et al., 2017) and thus be more sensitive to the intervention (Fallon et al., 2019). The order of testing was counterbalanced across drug manipulation, gender, and order of background foraging environment (rich-poor or poor-rich).

#### Statistical analyses

We used a hierarchical linear mixed-effects model (*fitlme* in MATLAB, The MathWorks; maximum likelihood estimation method) as our primary analysis method for both experiments, to account for between- and within-subject effects. All fixed effects of interest (patch type, environment, and, where applicable, dopamine) and their interactions were included, and the random effects structure was determined by systematically adding components until the AIC was minimized (Barr et al., 2013). Notably, the significance of any effects in all these models was the same as simpler models fitting only a random effect of subject. Significant model effects were also followed up with parametric tests (*t* tests and/or ANOVA).

Patch-leaving time was used as the dependent variable for analyses, as this was the primary behavioral measure from the experiment, and we predicted independent effects of changing patch type and background environment on this measure (based on MVT). It is worth noting that previous work has used the related variable, patch reward rate at leaving as the dependent variable (Constantino and Daw, 2015).

##### Study 1

LeavingTime=1 + patch×env + (1|sub) + (1|sub:patch) + (1|sub:env) + (1|sub:patch:env)

##### Study 2

LeavingTime=1 + patch×env×DA + (1|sub) + (1|sub:DA) + (1|sub:patch) + (1|sub:env) + (1|sub:patch:env:DA) where patch indicates patch type, env indicates background reward rate, DA indicates dopamine state, and sub indicates subject.

To avoid the potentially biasing effects of outlying data points on the primary analysis, we excluded, subject by subject, any trials in which the leaving time was >3 SDs from that individual's mean leaving time. Of note, this approach did not change the significance (or otherwise) of any reported results compared with analysis of the full dataset. Finally, the above linear mixed-effects models were also fitted using using patch reward rate at leaving time as the dependent variable instead of leaving time.

## Results

### Healthy human foragers are guided by MVT principles

**Figure 2.**
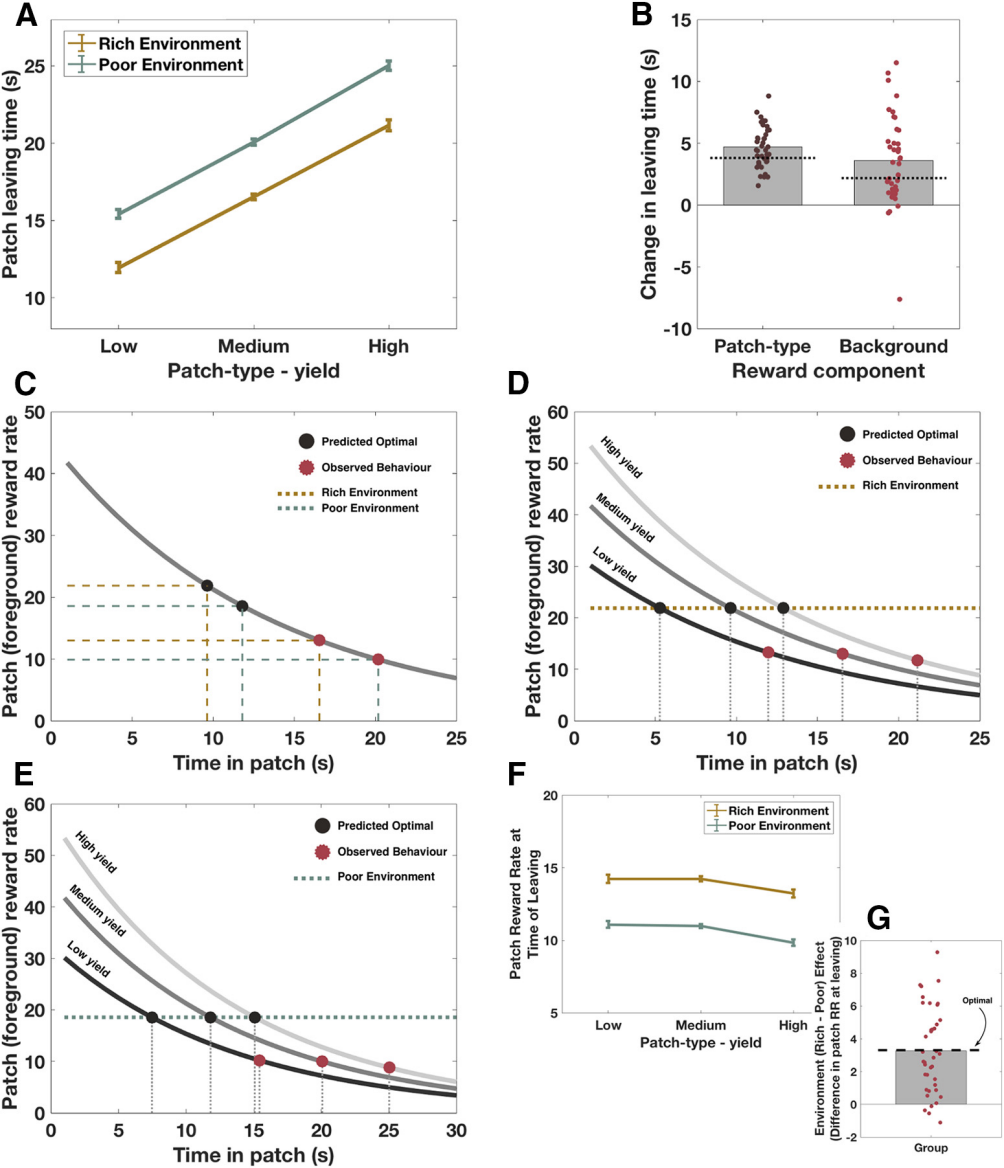
Healthy human foragers are guided by MVT principles. ***A***, Raw patch-leaving times. Participants (*N* = 39) left patches later when the background environment was poor, compared with rich (*p* < 0.00001), and when patches had higher, compared with lower yields (*p* < 0.00001), with no interaction between patch type and background environment (*p* = 0.2). ***B***, These effects of changing reward parameters were in the predicted direction, with participants leaving on average 4.7 s later as patch type varied, and 3.6 s later in poor compared with rich environments. There was more variation between individuals in the effects of changing background, compared with foreground, reward rates. Dashed lines indicate predicted (MVT) effects of changing reward rate on leaving time. ***C–E***, Participants showed a bias to remain in patches longer than predicted by MVT. Mean leaving time for each environment, collapsed across patch type, is shown in ***C***, whereas ***D*** and ***E*** demonstrate mean leaving times for each patch type in the rich and poor environments, respectively. ***F***, The foreground (patch) reward rate at which participants chose to leave each patch varied as a function of background environmental richness (rich vs poor). ***G***, The magnitude of this background environment effect was close to optimal (as predicted by MVT). Error bars indicate ± SEM.

Within MVT, foreground and background reward rates should have independent effects on how long an individual remains in a patch. People should leave low-yield patches sooner than high-yield patches, and patches in rich environments sooner than patches in poor environments. In line with these hypotheses, in Study 1, we found a main effect of patch type, as well as a main effect of background reward, but no interaction on participants' (*N* = 39) decisions about when to leave their current patch (patch type: *F*_(1,74.6)_ = 528, *p* < 0.0001; background: *F*_(1,37.5)_ = 40, *p* < 0.0001; patch type × background: *F*_(1,1929)_ = 1.6, *p* = 0.2; Table 1). Furthermore, behavior conformed to predicted directionality of these effects, with higher patch yield, and poor compared with rich background environment, both leading to later patch-leaving times (Fig. 2*A*,*B*).

**Table 1.** Linear mixed-effects models from each experiment with patch-leaving time as dependent variable

	PE	*T* statistic	*F* statistic	df*^a^*	*p*
Study 1					
Intercept	18.4	19.3	371	38.9	<0.00001
Patch type (P)	3.9	23.0	528	74.6	<0.00001
Environment (E)	1.8	6.3	40	37.5	<0.00001
P × E	0.1	1.3	1.6	1928	0.2
Study 2					
Patch type (P)	3.63	20.6	425	57.3	<0.00001
Environment (E)	0.76	4.1	16.9	28.1	0.0003
Dopamine (D)	−0.34	−1.37	1.86	29.3	0.18
E × D	−0.18	−2.29	5.22	200.1	0.023
P × E	0.01	0.17	0.03	197.1	0.86
P × D	−0.09	−1.14	1.29	186.6	0.26
P × E × D	−0.04	−0.56	0.31	186.4	0.58

*^a^*df was calculated using the Satterthwaite correction method.

### Are healthy people optimal foragers?

Although participants showed effects in the directions predicted by MVT, we wanted to know whether the magnitude of these effects conform to foraging theories, which stipulate the optimal time to leave each patch. Every individual showed a significant bias to remain longer across all patch types (across both environments) than optimal, on average leaving 8.0 s later than MVT predictions (*t*_(38)_ = 8.4, *p* < 0.001; Fig. 2*C–E*). However, it has been noted that humans, nonhuman primates, and other animals also show such a bias to stay (Nonacs, 2001; Stephens, 2008; Hayden et al., 2011; Constantino and Daw, 2015). This may relate to factors not modeled within the MVT, such as risk of predation when moving between patches, the possibility of organisms wishing to avoid a lower bound of energy stores (which could for example lead to starvation in certain contexts, e.g., when patches are sparsely distributed), other activities (e.g., grooming, finding a mate), and also potential neuro-computational limits (Bateson and Kacelnik, 1996; Nonacs, 2001; Stephens, 2008). However, it has been shown that patch-leaving behavior can be close to optimal once controlling for this bias, for example, by analyzing the relative changes across conditions, as we performed in this study (Hayden et al., 2011). Therefore, for each participant, we subtracted their own mean leaving time from each of their patch-leaving decisions, and calculated the magnitude of the background (poor - rich) and foreground (mean change between each patch type) reward rate effects (Fig. 2*B*,*G*).

MVT makes two core predictions about behavior as foreground and background reward rates change, which can be used to assess optimality of foraging behavior (independent to any systematic bias to remain in patches longer). First, in the background environments (poor vs rich), the foreground reward rate at leaving a given patch type should differ by the same amount. Second, foragers should adjust their leaving time as patch quality varies, such that the instantaneous reward at leaving is the same in each patch (for a given background). That is, within an environment, each patch should be left, regardless of its yield, when the rate at which milk is being accrued is the same.

Strikingly, participants varied their leaving times as background environment changed, such that the difference in reward rate between the two conditions was not significantly different from the predicted optimal difference (mean difference in reward rate at leaving = 3.33, actual difference between environments if behaving optimally = 3.30, *t*_(38)_ = 0.07, *p* = 0.95; Fig. 2*F*,*G*). In contrast, the foreground reward rate at patch leaving did vary across patch type (*F*_(1.5,42)_ = 6.73, *p* = 0.005, repeated-measures ANOVA). Although the instantaneous reward rate on leaving low- and medium-yield patches did not differ (mean difference = 0.06, *t*_(37)_ = 0.2, *p* = 1), participants remained in high-yield patches until the instantaneous reward rate was lower compared with both medium-yield (mean difference = 1.1, *t*_(37)_ = 3.8, *p* = 0.002), and low-yield patches (mean difference = 1.1, *t*_(37)_ = 2.6, *p* = 0.04; Fig. 2*F*; Table 2). This means that participant behavior on average was not completely optimal with respect to MVT predictions.

**Table 2.** Linear mixed-effects models for each experiment with patch reward rate at leaving time as the dependent variable

	PE	*T* statistic	*F* statistic	df^a^	*p*
Study 1					
Intercept	2.4	34	1128	38.9	<0.00001
Patch type (P)	−0.05	−4.0	16	74.6	0.0002
Environment (E)	−0.1	−6.3	40	37.5	<0.00001
P × E	−0.008	−1.1	1.1	1927	0.3
Study 2					
Patch type (P)	-0.03	−2.3	5.1	57.3	0.03
Environment (E)	−0.06	−4.1	16.9	28.1	0.0003
Dopamine (D)	−0.03	−1.37	1.9	29.3	0.18
E × D	−0.01	−2.29	5.22	200.1	0.023
P × E	<−0.001	−0.1	0.01	197.1	0.92
P × D	−0.006	−1.14	1.3	186.6	0.26
P × E × D	−0.003	−0.56	0.31	186.4	0.58

*^a^*df was calculated using the Satterthwaite correction method.

There was no systematic difference in leaving times across the experiment. We compared leaving times in the first and second half of the experiment for each person, for the two most commonly encountered conditions (high-yield/rich environment and low-yield/poor environment), and found no significant changes for either condition: high/rich, mean difference (first half – second half) = 0.7 ± 4.0 s, *t*_(38)_ = 0.97, *p* = 0.33; low/poor, mean difference = 1.1 ± 3.9 s, *t*_(38)_ = 1.8, *p* = 0.08.

**Figure 4.**
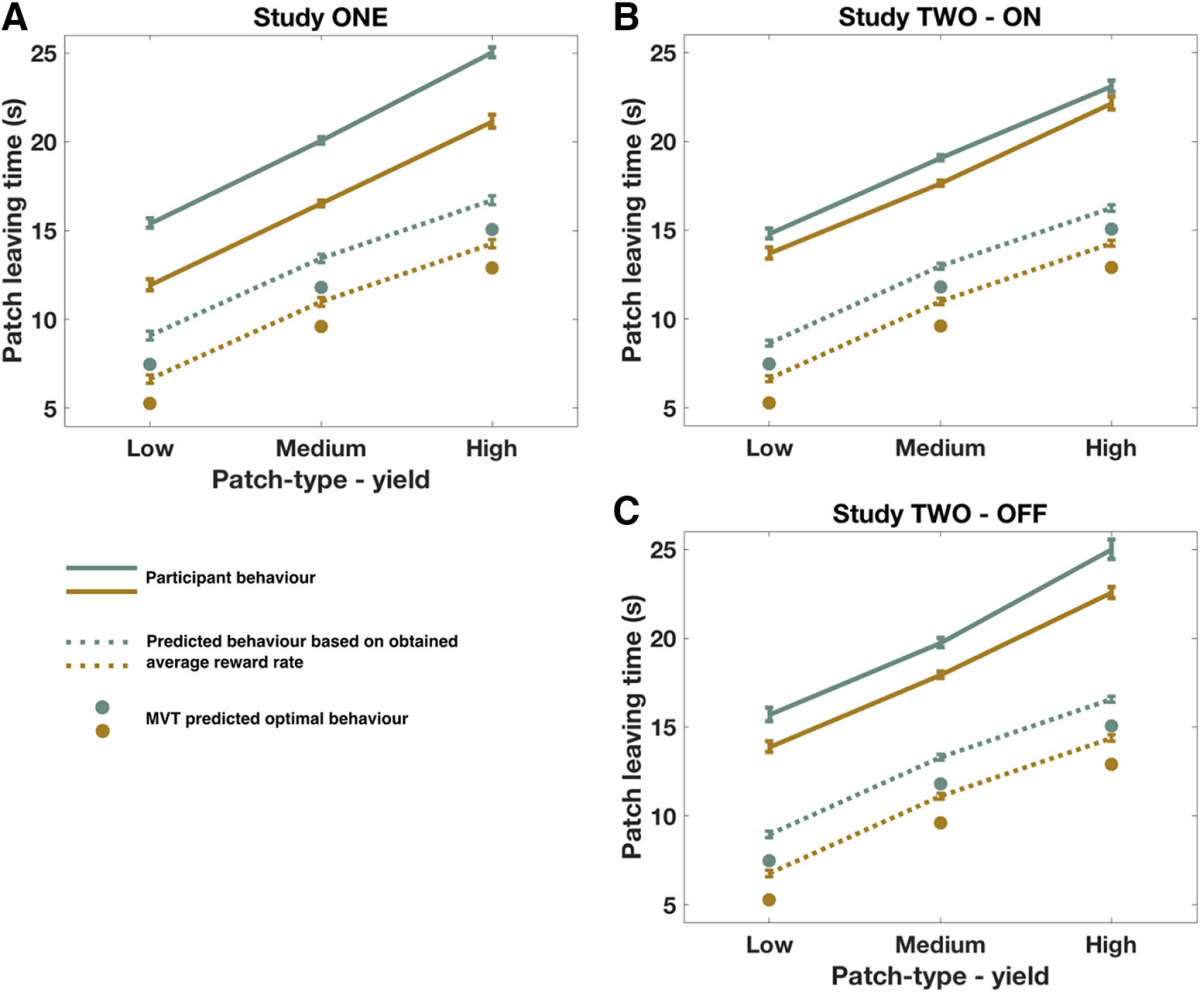
Patch-leaving times: observed, and predicted based on MVT. ***A–C***, Although predicted leaving times based on actual long-run background reward rate were later than optimally predicted by MVT, actual leaving times were still significantly later. Dots represent MVT predicted optimal leaving time. Dotted lines indicate predictions based on actual long-run background reward rate. Lines indicate actual behavior. Green represents poor environment. Gold represents rich environment.

Finally, to investigate whether the observed overstaying tendency could be a consequence of suboptimal leaving decisions reducing the perceived background reward rate (and thus, in accordance with MVT, leading to later leaving times than predicted), we calculated for each participant the predicted patch-leaving times based on their actually obtained long run background reward rate. Across participants, the average obtained background reward rate in both the rich and poor environments was significantly lower than the maximum available assuming optimal behavior (rich environment: obtained = 19.8 ± 1.9, max optimal = 21.9, *t* test for difference: *t*_(38)_ = 6.5, *p* < 0.0001; poor environment: obtained = 16.5 ± 1.5, max optimal = 18.6, *t* test for difference: *t*_(38)_ = 8.5, *p* < 0.0001). These background reward rate metrics translated into predicted patch-leaving times which were 1.4 s later from each patch in the rich environment, and 1.6 s later from each patch in the poor environment. Despite this adjustment, however, participants still left on average 5.9 s later than predicted from patches in the rich environment and 7.1 s later than predicted from patches in the poor environment, meaning that the observed tendency to overstay was not simply a result of variations in participants' actual performance reducing the perceived background reward rate (see Fig. 4*A*).

In summary, across participants patch-leaving behavior as patch type changed was not optimal, whereas on average participants adjusted leaving times in response to changes in the background environment in a manner that closely matched the actual changes in background reward rate. They also adjusted their leaving behavior such that the reward rate at leaving did not differ between low- and medium-yield patches, although they tended to leave high-yield patches later (i.e., after patch reward rate had dropped further). Thus, human behavior on this task somewhat conformed to MVT principles but was not completely optimal. However, our results suggest that, when people do have to make decisions about when to leave a location to collect rewards elsewhere, the patch and environment effects on behavior are in the same direction as MVT would predict. This is despite no instructions of what pattern of behavior would maximize rewards in the task.

### Cabergoline alters the use of background reward information to guide patch leaving

Having demonstrated that healthy human patch-leaving behavior is not optimal, but conforms to the broader principles of MVT, particularly in response to changes in background reward rate, we next examined whether dopamine modulates the effect of background reward rate (environment) on patch-leaving behavior. Using a within-subjects design, in Study 2, leaving times for 29 healthy older people on placebo or following administration of the D2 receptor agonist cabergoline were analyzed using a LME model.

**Figure 3.**
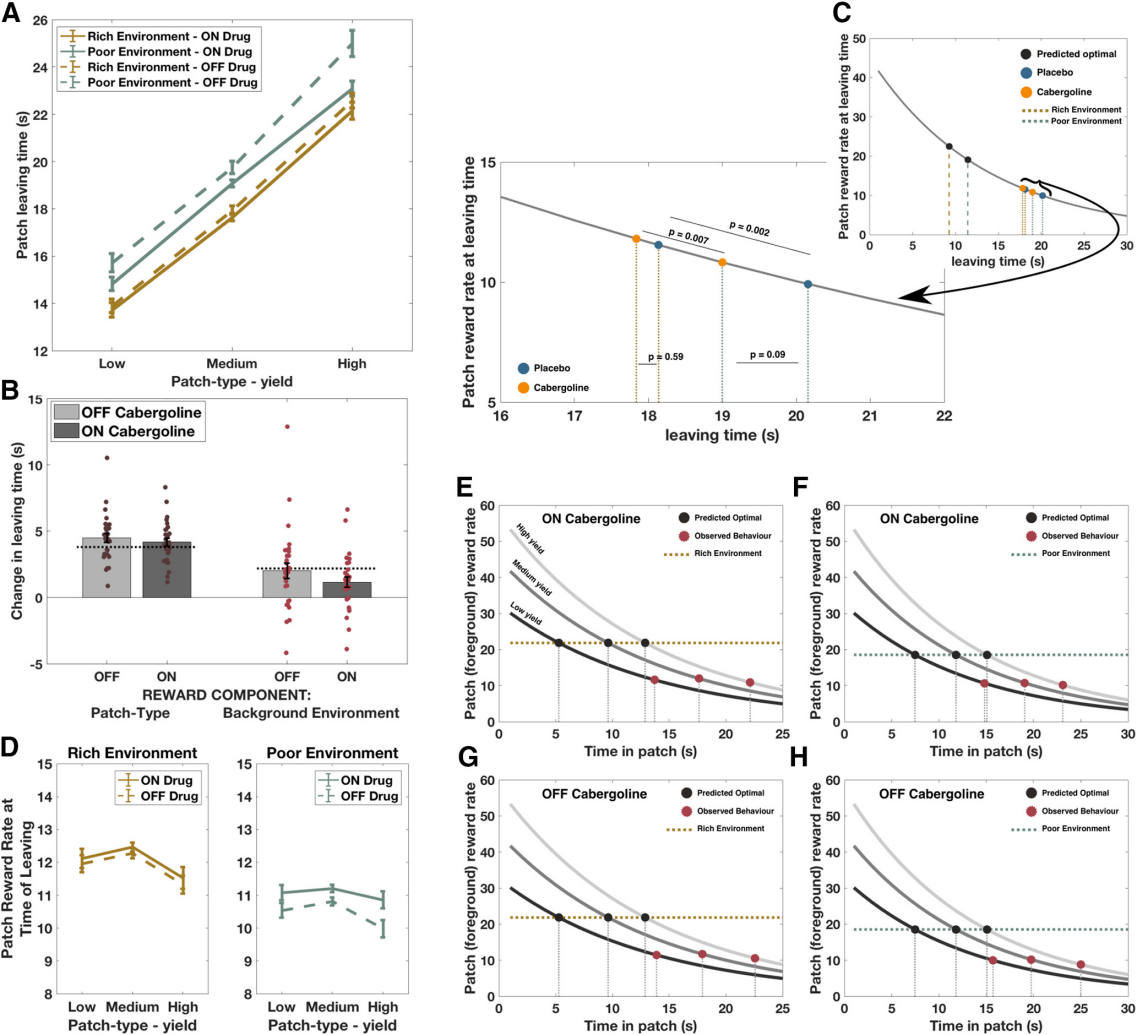
Cabergoline alters use of background reward information to guide patch leaving. ***A***, Mean patch-leaving times for each patch type, split by environment and drug state. ***B***, There was a significant interaction between drug and background (environment) reward rate on leaving time, with a reduced effect of background environment ON cabergoline compared with OFF (*p* = 0.023). In contrast, there was no significant interaction between drug and the effect of changing foreground (patch type) reward on patch leaving (*p* = 0.26). Black dotted lines indicate the predicted magnitude of effect of changing patch type and background environment, based on the MVT. ***C***, Instantaneous patch reward rate at time of leaving, collapsed across patch types. Participants showed a significant bias to leave all patch types later than optimally predicted. The effect of cabergoline was mainly driven by participants leaving patches in the poor environment earlier ON drug and, therefore, when the current patch reward rate was higher (inset). ***D***, Alternative representation of data, plotting instantaneous reward rate at time of leaving each patch type in each environment, ON and OFF cabergoline. ***E***–***H***, Relationship between patch-leaving time and patch reward rate for each condition, ON and OFF cabergoline. *N* = 29, comparisons are within-subject. Error bars indicate ± SEM.

First, the main effects reported in Study 1 were replicated. Both patch type and background (environment) reward rate significantly influenced patch-leaving time, and there was no interaction between the two (patch type: *F*_(1,57)_ = 425, *p* < 0.0001; background: *F*_(1,28)_ = 16.9, *p* = 0.0003; patch type × background: *F*_(1,197)_ = 0.03, *p* = 0.86; Fig. 3*A*,*B*). Furthermore, the magnitude of effect of both background reward rate and patch type on leaving time did not significantly differ between the young and older groups [mean difference (young – old) patch type = 0.2 s, *t*_(66)_ = 0.49, *p* = 0.62; mean difference (young – old) background = 1.2 s, *t*_(66)_ = 1.77, *p* = 0.08].

There was a significant interaction between drug state and the effect of background reward rate on leaving time (*F*_(1,200)_ = 5.22, *p* = 0.023; Table 1). When ON cabergoline, people were less sensitive to the difference between poor and rich environments than when OFF drug, although they still showed a significant effect of background environment both ON and OFF the drug (Fig. 3*A*,*B*). *Post hoc* analysis suggests that this interaction was driven by people leaving patches in the poor environment much earlier ON cabergoline than OFF, but only leaving patches in the rich environment slightly earlier ON compared with OFF (mean difference (OFF – ON) poor environment = 1.2 s, *t*_(28)_ = 1.75, *p* = 0.09; mean difference (OFF – ON) rich environment = 0.3 s, *t*_(28)_ = 0.59, *p* = 0.56; Fig. 3*C*,*D*).

We hypothesized that modulating dopamine levels would not alter the effect of changing patch types on patch leaving, if manipulating tonic levels predominantly affects the processing of average reward rates. In line with this hypothesis, there was no significant drug × patch interaction (*F*_(1,187)_ = 1.29, *p* = 0.26): cabergoline did not lead to a significant change in the way participants used foreground reward rate information to guide leaving decisions (Fig. 3*B*). There was also no statistically significant difference in leaving times overall on drug compared with placebo (mean difference = 0.73 s, *F*_(1,29)_ = 1.86, *p* = 0.18), nor did the reward rate at leaving vary as a function of drug state (mean difference = 0.39, *t*_(28)_ = 0.8, *p* = 0.41). Furthermore, these results were consistent regardless of whether leaving times, or patch reward rate at leaving time, was analyzed as the dependent variable (Table 2).

As would also be predicted within MVT, there was no interaction in leaving times between patch type and background reward rate. Moreover, the observed drug × background reward rate interaction was present across all patch types, with no three-way interaction (*F*_(1,186)_ = 0.31, *p* = 0.58). All of these results remain significant after controlling for weight, height, and BMI. Although the experiment was designed to minimize the effects of any learning, because the dopaminergic manipulations could in theory lead to differential learning effects between states, we analyzed the data from experiment two for session or order effects. The inclusion of session (first or second) worsened model fit (ΔBIC = 7.6), and the parameter estimate for session effect was not significant (PE = −0.16, *F*_(1,29)_ = 0.36, *p* = 0.56). Similarly, including order (the session × drug interaction) also worsened model fit (ΔBIC = 14.2), and again this term was not significant (PE = 0.87, *F*_(1,29)_ = 1.4, *p* = 0.25). Therefore session and order effects were not included in the final model. The inclusion of these effects did not change the significance (or otherwise) of the other model terms. There was also no evidence of a systematic shift in patch-leaving behavior across the course of each session as a function of drug state. We calculated, for each subject, the mean leaving time in the first and second half of each session (ON and OFF) for the two conditions with the highest number of trials (high-yield patch in rich environment and low-yield patch in poor environment). Using this metric, the mean difference in leaving time across the experiment was not significantly different between the cabergoline and placebo conditions (Mean Difference_PLAC-CAB_ = −0.4 s, *t*_(28)_ = 0.78, *p* = 0.44).

Finally, to test whether learning during the task was influencing behavior as a function of drug state, we fitted a model for the cabergoline data that included the previous trial outcome (reward obtained on the previous trial) as a predictor of patch-leaving time on the subsequent trial, and compared this with our primary model that did not include this metric (Garrett and Daw, 2019). Although the parameter estimate for the term “reward on previous trial” was significant (*F*_(1,2776)_ = 12, *p* = 0.001), inclusion of this term led to a worsening of model fit, as measured by either Bayesian Information Criterion (ΔBIC = 50) or Aikake Information Criterion (ΔAIC = 2.1). To further investigate the potential for dopaminergic state to be changing task performance by an interaction with learning, we also calculated, for each participant, the difference in the parameter estimate for “reward on previous trial” between the ON and OFF states. We found no evidence of a systematic change based on previous trial (mean change [ON vs OFF] = −0.1, *t*_(28)_ = 1.19, *p* = 0.25). These results suggest that participants' behavior was not systematically changing from one patch to the next based on the rewards received in the last patch as a function of dopamine state.

As observed in Study 1, people showed a significant bias to remain in all patch types longer than expected (Fig. 3*E–H*). Again, this observation was not explained by predicting optimal behavior based on actually obtained long-run background reward rate (rather than MVT predicted optimal); on average, people still left 6.8 and 7.3 s later than predicted from each patch type in the ON and OFF state, respectively (*p* < 0.0001 for each comparison; Fig. 4*B*,*C*).

Could participants be paying less attention when off medication? We analyzed leaving time variability to examine whether participants' decisions were noisier as a function of drug state. There was no significant difference in the variance of each participant's decisions between placebo and cabergoline conditions (Mean Difference_PLAC-CAB_ = 0.31, *t*_(28)_ = 1.34, *p* = 0.19). Therefore, cabergoline had a specific rather than general effect on patch-leaving behavior, altering only the influence of background reward rate on leaving time.

## Discussion

When to move on and leave a rewarding activity or location is an essential decision problem for animals and humans alike. Here, we show that humans, both young and old, make dynamic foraging decisions that, although not optimal, broadly conform to ecological principles captured by MVT (Charnov, 1976; Stephens and Krebs, 1986). Furthermore, dopaminergic D2 receptor activity may play a crucial role in modulating such decisions. Specifically, the findings support the view that dopamine plays an important role in signaling the average value of alternative locations, influencing dynamic decisions of when to move on. Administration of cabergoline altered the effect of background, but not foreground, reward rate changes on patch-leaving times. In particular, this interaction between cabergoline and background reward rate was driven mainly by people leaving all patches in poor environments earlier.

The results provide new evidence for the role of dopamine in decision-making. Manipulation of dopamine levels modulated the influence of background reward rate on dynamic decisions about when to switch behavior. Specifically, ON cabergoline people tended to leave all patch types in the poor environment earlier than when OFF drug. In contrast, in the rich environment, there was a much smaller change in leaving times between the ON and OFF drug states. The drug manipulations used here putatively alter tonic dopamine levels (Brooks et al., 1998), a component of the dopaminergic neuromodulatory system which has been ascribed, in the context of motor responses, a role in signaling background reward rates (Niv et al., 2007; Hamid et al., 2016). Of course, in this study, we were not able to measure firing rates of dopamine neurons, and the relationship between firing rates, dopamine availability, and dopamine receptor activity is far from clear (Mohebi et al., 2019). Nevertheless, some existing evidence suggests that tonic dopamine levels encode information about background reward rate, and therefore the opportunity cost (alternatives that are foregone) of chosen actions (Niv et al., 2007; Guitart-Masip et al., 2014).

Much of the previous research in this area has used bandit-type designs to better understand dopaminergic functions, which although useful, may not always reflect real-world problems. Furthermore, in such experiments, foreground and background reward rates can become correlated, such that the value of exploring alternatives has an instrumentally predictive value of obtaining an immediate (foreground) reward (Daw et al., 2006; Kayser et al., 2015; Westbrook and Frank, 2018). However, in ecological settings, choices to “leave” a patch and explore are not choices between two stimuli with a predictive value but instead involve traveling to obtain rewards elsewhere. Thus, rewards available in a patch can be orthogonal to the environment one is in. Using an MVT-inspired paradigm, we showed that D2 manipulation impacts on background reward rates. This parallels results from a recent study, which used a different patch-leaving design, administered to people with Parkinson's disease ON and OFF their normal dopaminergic medications, to test a similar hypothesis (Constantino et al., 2017). Patients left patches at lower reward rate thresholds (i.e., stayed in patches for longer) when OFF medications, consistent with a lower estimation of the background reward rate in a dopamine-depleted state. In the current study, we show such effects are specifically linked to D2 receptors in healthy people, suggesting that D2-mediated pathways may be of particular importance for signaling such contextual reward information (Beaulieu and Gainetdinov, 2011). When D2 receptors were stimulated (ON state), people left patches earlier (at a higher foreground reward rate) in the poor environment, consistent with an increase in perceived richness of the environment. This effect was not observed in the rich environment, possibly because of a ceiling effect (D2 stimulation having reduced effect on behavior when reward rates were already high); future research using multiple drug doses or multiple environments could investigate this issue further. Overall, while dopaminergic stimulation may increase the vigor of movements or exploratory binary choices, in more abstract, ecological decision settings, it serves to increase the perceived environmental richness, setting a higher threshold reward rate of when to leave.

Importantly, these results appear to be driven by changes in sensitivity to the background reward rate, rather than alternative explanations. First, patch rewards were constantly being accrued, rather than stepped changes, as has been used in previous studies (Hutchinson et al., 2008; Constantino and Daw, 2015). This approach has the advantage of minimizing the use of simple heuristics to guide decisions while leaving the dependent variable approximately normally distributed. It should be noted, though, that mathematically MVT principles hold for both discreet and continuous patch-leaving designs. Second, variance in patch-leaving times did not change as a function of drug state. This makes it unlikely that the results can be explained by a confounding factor, such as reduced attention. Third, as participants were explicitly informed of the current environment in which they were in, had experienced the different background reward rates in a training phase, and did not systematically alter patch-leaving behavior across the experiment, it is unlikely that the results could be explained by differences in learning as a function of drug. Finally, although the observed results could theoretically be explained by dopaminergic stimulation reducing subjects' estimation of current patch reward rate (instead of altering background reward rate appraisal), the lack of main effect of dopamine on leaving time and the absence of change in behavior in patches embedded within the rich environment makes this unlikely.

One further possible interpretation is that differential learning of background reward rates within the training phase (ON vs OFF drug) could have influenced subsequent patch-leaving behavior. Dopamine has a long history of being linked to learning through reward prediction errors (Frank et al., 2004; Daw and Doya, 2006; Schultz, 2007). In this study, we controlled for such effects by showing absence of order effects in behavioral data, by explicitly training participants on the environmental richness, as well as instructing them of this at all times while in patches. However, it is plausible that participants could have been poorer at learning the average reward value in each environment when OFF drug in the training session, due to changes in how prediction errors were signaled. Such an effect seems unlikely given the absence of an effect of cabergoline on rich environment leaving times, suggesting that participants were able to learn the rich environment reward rates ON or OFF the drug. However, even if driven by a failure to learn, our results show the consequences: poor environments are treated as richer, leading to reduced patch residency times when ON cabergoline. Furthermore, although not the focus of the current manuscript, the overlapping and dissociable effects of the dopaminergic system on both reward motivated behavior and learning are an evolving research area (Cools et al., 2011; Berke, 2018) that the use of foraging-style tasks may be particularly important for advancing understanding of (Constantino and Daw, 2015).

Our results highlight that human foraging behavior broadly conforms to the principles of MVT, although it is suboptimal (Charnov, 1976; Pearson et al., 2014). This accords with earlier field work in behavioral ecology (Stephens and Krebs, 1986; Pearson et al., 2014) and anthropology (Smith et al., 1983; Metcalfe and Barlow, 1992) literature, and more recent work beginning to explore the neural basis of such decisions (Hayden et al., 2011; Kolling et al., 2012; Constantino and Daw, 2015). In the current study, the use of a foraging framework informed by MVT enabled us to dissociate the effects of reward rates on different time scales, which are often correlated in reinforcement learning-based manipulations of average reward rates (Niv et al., 2007; Mobbs et al., 2018). Specifically, it allowed us to examine whether dopaminergic modulations impacted on one, either, or both reward components, with our results showing an effect of cabergoline only on the background rate.

From a clinical perspective, these findings may be significant when considering mechanisms underlying common disorders of motivated behavior, such as apathy (Le Heron et al., 2019). Apathy is often associated with disruption of mesolimbic dopaminergic systems (Santangelo et al., 2015), and, at least in some cases, can be improved with D2/D3 receptor agonists (Adam et al., 2013; Thobois et al., 2013). Accumulating evidence demonstrates altered reward processing in patients with apathy (Strauss et al., 2014; Le Heron et al., 2018a), and it is plausible, although as yet untested, that chronic underestimation of background environment reward leads to a state where it is never “worth switching” from a current activity, even if this activity is very minimal. Future work could profitably explore this hypothesis.

Recent theoretical accounts of decision-making have called for a shift to more ecologically derived experiments to investigate the mechanisms of this fundamental neural process (Pearson et al., 2014; Mobbs et al., 2018). The current results highlight the utility of such an approach, demonstrating a role for D2 activity in signaling the average background reward rate during foraging. It links basic ecological models of animal behavior to a mechanistic understanding of human decision-making, highlighting the specific influence of dopaminergic systems as people decide when to move on as they pursue rewards in their environment.
